# Supplementary material for: The Deep Generative Decoder: MAP estimation of representations improves modelling of single-cell RNA data
Source: Bioinformatics. 2023 Aug 12;39(9):btad497. doi: 10.1093/bioinformatics/btad497 (PMC10483129; doi:10.1093/bioinformatics/btad497)
Supplement: btad497_Supplementary_Data [file btad497_supplementary_data.pdf]

Supplementaries

The softball prior

In the softball prior used on the GMM means $\boldsymbol{\mu}$, the means are initialized as

$$\begin{aligned} \boldsymbol{\mu} &= \text{scale} \times \mathbf{l} \times \frac{\mathbf{u}}{\|\mathbf{u}\|} \\ \text{with } \mathbf{u} &\sim \mathcal{N}(0,1) \text{ and } \mathbf{l} \sim \mathcal{U}(0,1)^{m^{-1}}. \end{aligned} \tag{5}$$

The log-probability of this prior is given as

$$\begin{aligned} \log P(\boldsymbol{\mu}) &= A - \log\left(1 + e^{\text{sharpness}\left(\frac{\|\boldsymbol{\mu}\|}{\text{scale}} - 1\right)}\right) \\ \text{with } A &= \log\Gamma(1 + 0.5m) - m(\log(\text{scale}) + 0.5\log\pi) \end{aligned} \tag{6}$$

The normalization constant can be understood as the volume of the m-ball and represents an approximation of the true normalization constant.

Figures and tables

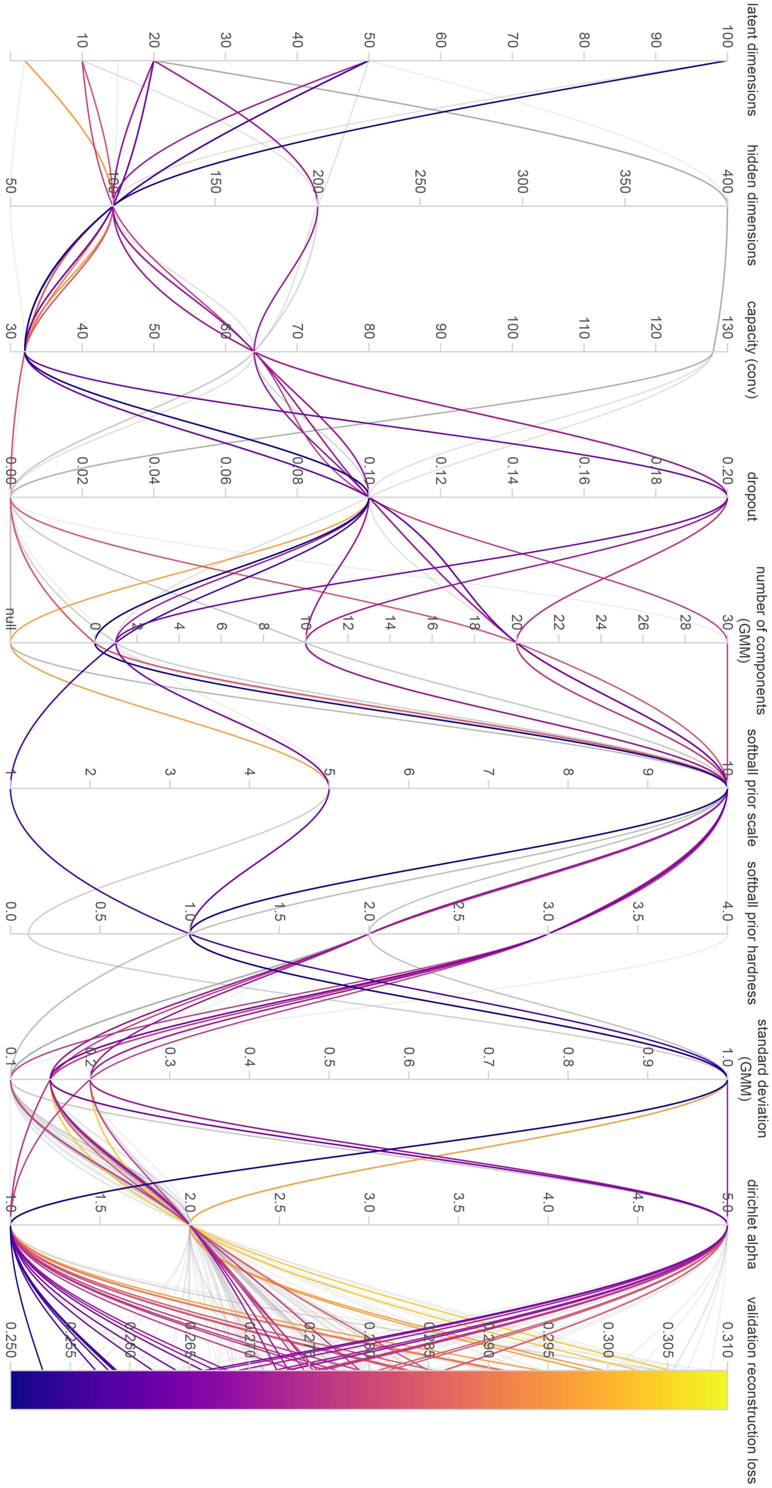

Fig. 1: **Fashion-MNIST hyperparameter search.** The parallel coordinate plot from the corresponding wandb (Biewald, 2020) project shows combinations of hyperparameters (all coordinates of the plot except the last) and the resulting models' reconstruction performance on the validation set. Each model is represented by a line colored by the validation reconstruction loss (BCE). A total of 132 different models were tested.

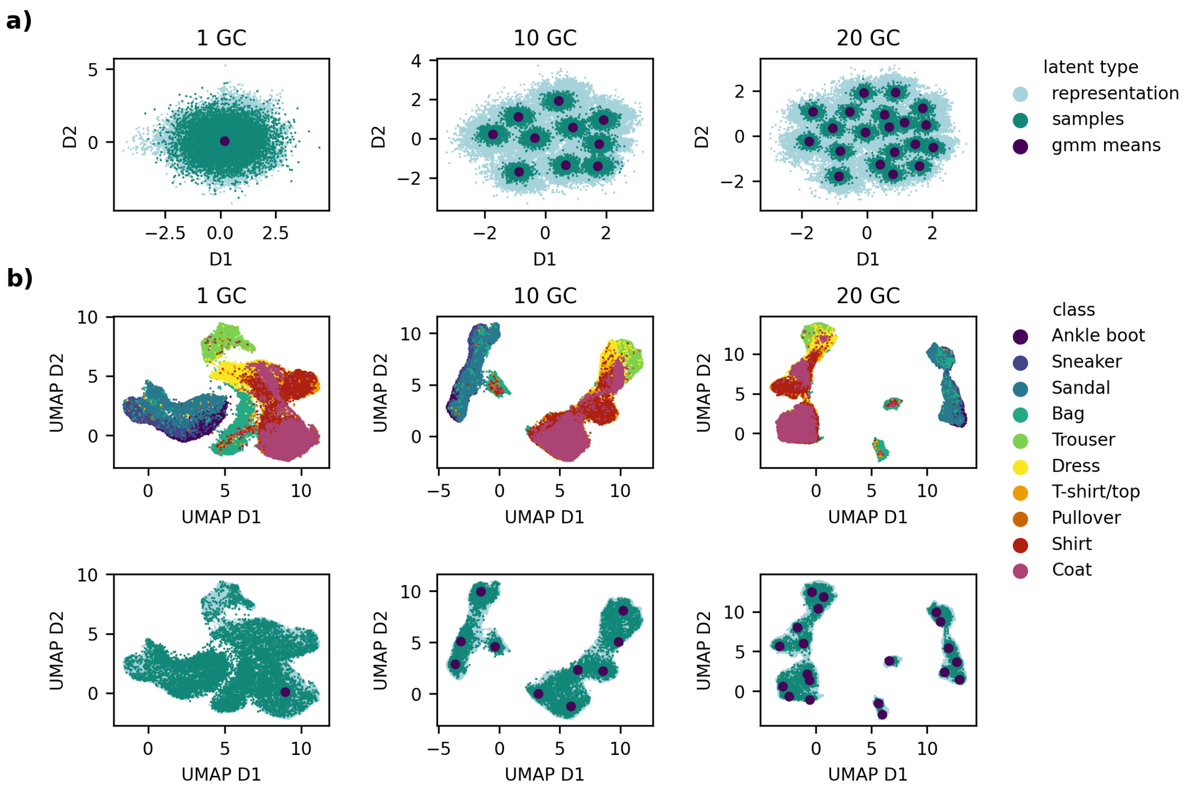

Fig. 2: **Latent spaces for varying numbers of Gaussian components and latent dimensionalities trained on Fashion-MNIST. a)** DGDs with a 2-dimensional latent space are trained with 1, 10 and 20 Gaussian components (GC). Latent points are colored by their type. This refers to whether they are learned representations, samples drawn from the GMM or component means. **b)** UMAP projections of 20-dimensional latent spaces with 1, 10 and 20 Gaussian components. The top row is colored by sample class, the bottom by latent point type as in a).

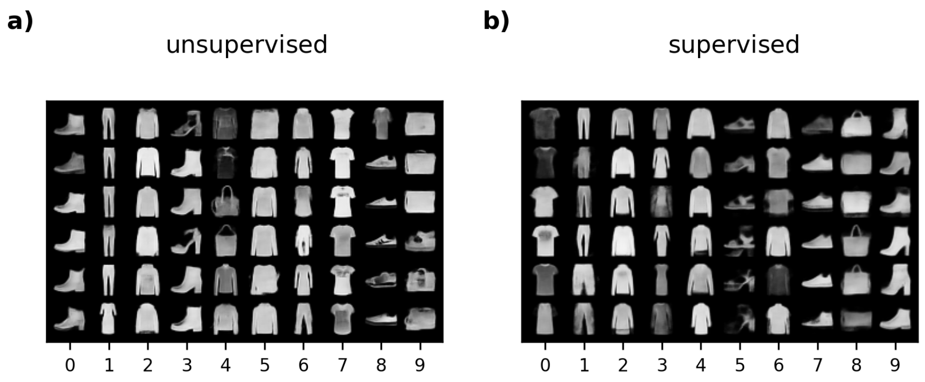

Fig. 3: **Unsupervised and supervised learning of representation and GMM for Fashion-MNIST.** DGDs with a 20-dimensional latent space are trained with 10 Gaussian components in a **a)** unsupervised and **b)** supervised manner. For each model, 6 reconstructed samples are shown (row-wise) for each of the 10 components, indicated by the component ID below.

Model	Latent	BCE loss	Shapiro-Wilk test statistic	ARI	FID score
DGD	20	0.2629 ± 0.001	0.9939	0.21	37.51
DGD	50	0.2578 ± 0.009	0.9774	0.14	74.34
DGD	100	0.2578 ± 0.009	0.9414	0.13	73.22
VAD	20	0.2615 ± 0.001	0.9962	0.38	44.15
VAD	50	0.2575 ± 0.009	0.9898	0.32	52.95
VAD	100	0.2583 ± 0.001	0.9543	0.31	46.37
VAE	20	0.2798 ± 0.001	0.7533	0.27	33.87
VAE	50	0.2808 ± 0.001	0.4431	0.29	34.47
VAE	100	0.2804 ± 0.001	0.2999	0.36	33.01

Table 1. Metrics of the quantitative analysis of DGD, VAD and VAE trained and evaluated on Fashion-MNIST. The BCE loss represents the averaged Binary Cross-Entropy loss of the reconstructed images. The lower it is, the better. The Shapiro-Wilk test statistic describes the Normality of the latent representation. The test statistic lies within the range of 0 to 1, the higher the better.

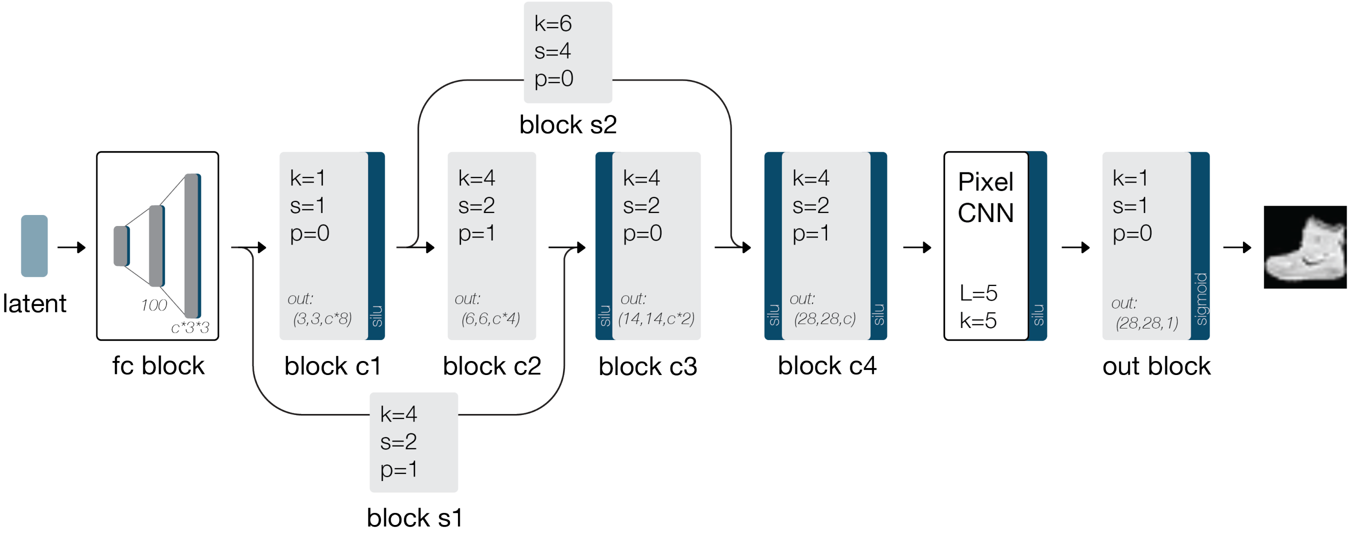

Fig. 5: **Schematic of the Fashion-MNIST decoder architecture.**

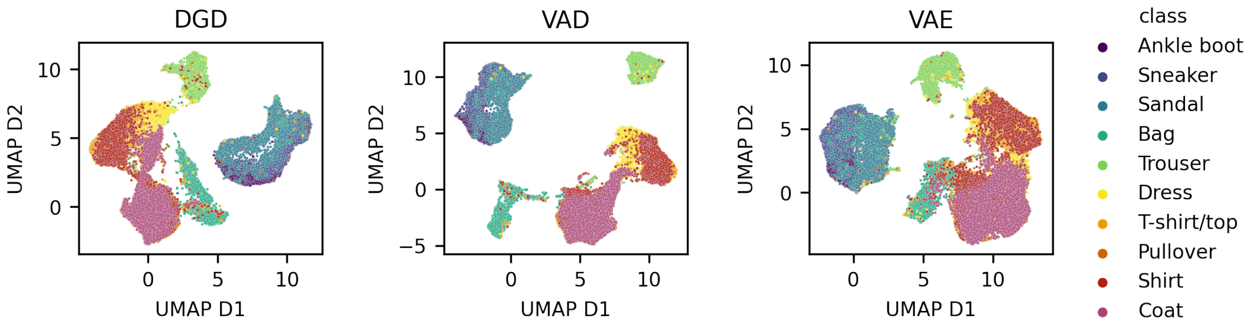

Fig. 4: **Fashion-MNIST latent space visualizations of DGD, VAD and VAE train representations.** Representations are visualized in the first two UMAP dimensions and samples are colored by the class label.

Table 2. Comparison of performance metrics for scDGD and scVI on the 1.3 million mouse brain cells data. Model names are indicated on the left. Performance metrics computed are indicated as the remaining columns. The NLL refers to the negative log-likelihood of the negative binomial distribution which models the counts in all methods. The best values for each metric are highlighted in bold.

Model	ARI	NLL	RMSE	Run time (hours)	Epochs	Resources (max)
scDGD	**0.351**	5382.60 ± 4.97	0.6153 ± 0.0035	25.82	800	53.1 GB (CPU), 9.1 GB (GPU)
scVI	0.279	5377.91 ± 4.94	**0.5622 ± 0.0021**	**13.81**	400	124.5 GB (CPU), 1.7 GB (GPU)

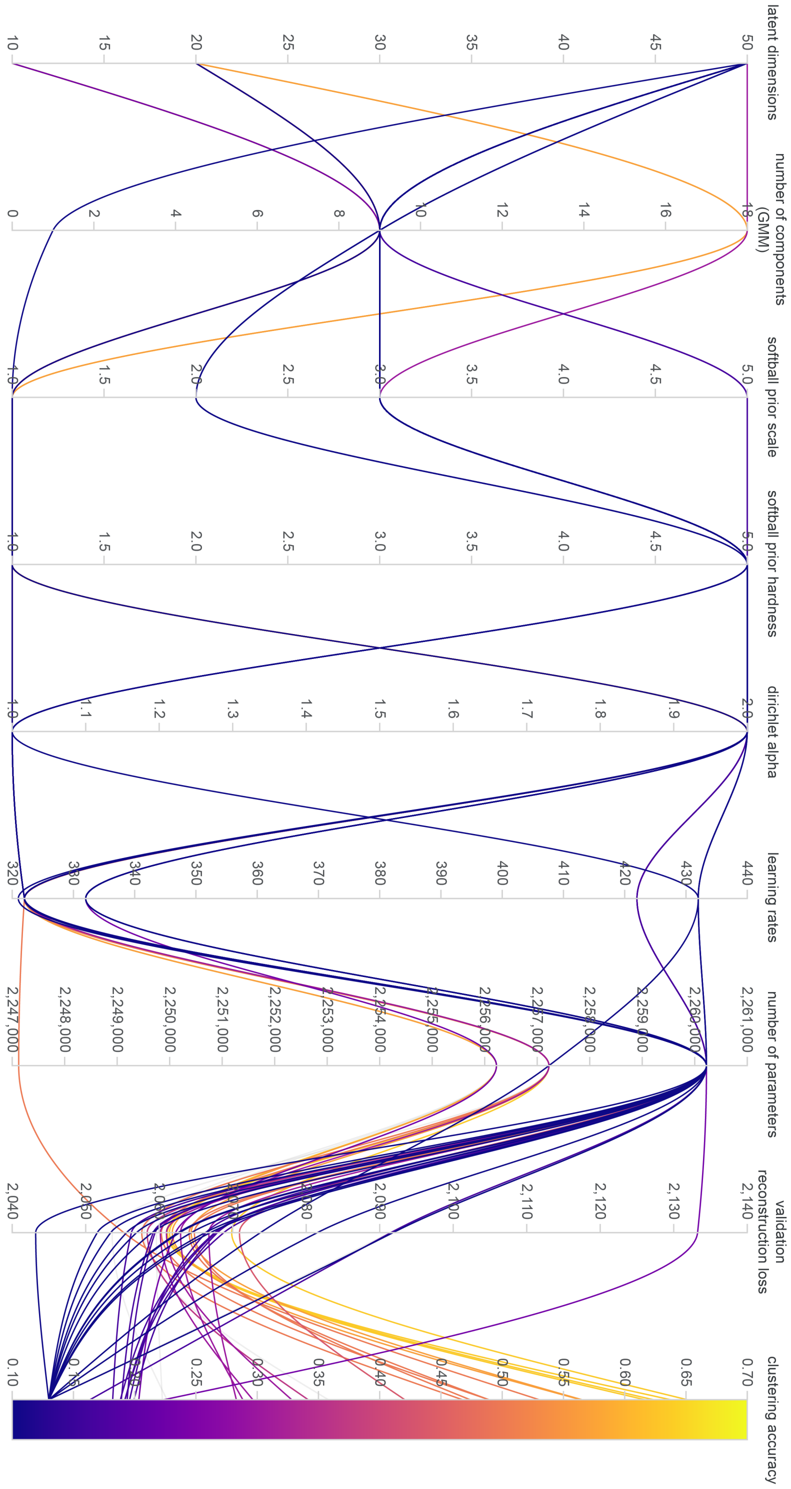

Fig. 6: **Single-cell DGD hyperparameter search.** The parallel coordinate plot from the corresponding wandb (Biewald, 2020) project shows combinations of hyperparameters (all coordinates of the plot except the last) and the resulting models' reconstruction performance on the validation set, as well as the clustering accuracy. Each model is represented by a line colored by the clustering accuracy with respect to the cell types of the data. A total of 67 different models were tested.

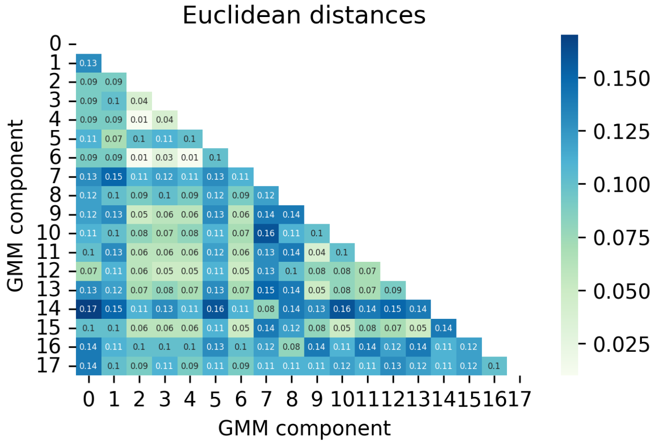

Fig. 7: **Euclidean distances between GMM component means of the 18-component scDGD.** Heatmap of the Euclidean distances between GMM component means.
